# Decision Support System for Variable Rate Irrigation Based on UAV Multispectral Remote Sensing

**DOI:** 10.3390/s19132880

**Published:** 2019-06-28

**Authors:** Xiang Shi, Wenting Han, Ting Zhao, Jiandong Tang

**Affiliations:** 1 College of Mechanical and Electronic Engineering, Northwest A & F University, Yangling 712100, China; 2 Key Laboratory of Agricultural Internet of Tings, Ministry of Agriculture, Yangling 712100, China; 3 Institute of Soil and Water Conservation, Northwest A & F University, Yangling 712100, China; 4 College of Enology, Northwest A & F University, Yangling 712100, China; 5 College of Water Resources and Architectural Engineering, Northwest A & F University, Yangling 712100, China

**Keywords:** decision support system (DSS), variable rate irrigation (VRI), fuzzy logic system, UAV multi-spectral image, duty-cycle control map

## Abstract

Rational utilization of water resources is one of the major methods of water conservation. There are significant differences in the irrigation needs of different agricultural fields because of their spatial variability. Therefore, a decision support system for variable rate irrigation (DSS-VRI) by center pivot was developed. This system can process multi-spectral images taken by unmanned aerial vehicles (UAVs) and obtain the vegetation index (VI). The crop evapotranspiration model (ET_c_) and crop water stress index (CWSI) were obtained from their established relationships with the VIs. The inputs to the fuzzy inference system were constituted with ET_c_, CWSI and precipitation. To provide guidance for users, the duty-cycle control map was outputted using ambiguity resolution. The control command contained in the map adjusted the duty cycle of the solenoid valve, and then changed the irrigation amount. A water stress experiment was designed to verify the rationality of the DSS-VRI. The results showed that the more severe water stress is, the more irrigation is obtained, consistent with the expected results. Meanwhile, a user-friendly software interface was developed to implement the DSS-VRI function.

## 1. Introduction

China is a country with a water shortage, especially in its northwest regions. The shortage of water resources restricts agricultural development in many provinces, while the need for food has dramatically increased with the growth of the population. Agriculture is facing more serious challenges and requires effective ways for water-saving irrigation. Variable rate irrigation (VRI) is distinguished from traditional precision irrigation and provides such a solution. Previous VRI research has focused on quantitative decision-making models, partition management, decision support systems (DSS) and variable outputs [1]. There among, the quantitative decision-making model and partition management are key parts of VRI, variable output plays the role of executor, and DSS connected all parts of VRI. 

The DSS has been discussed as a popular topic in the VRI system. Navarro [2] developed a DSS for estimating weekly irrigation needs on the basis of soil measurements and climatic variables which were gathered by several autonomous nodes deployed in farmland. Nain [3] used a DSS to generate suitable decision outputs for irrigation and fertilization in hilly regions. Miller [4] developed a DSS, which uses the Natural Resources Conservation Service Gridded Soil Survey Geographic Database to estimate water capacity available for root zone, and determines irrigation amount based on the estimates. Besides, AgroClimate is a DSS tool for improving the efficiency of irrigation water usage (http://mz.agroclimate.org/) with its irrigation decision made on the basis of daily crop evapotranspiration calculated by the Hargreaves equation and crop coefficients [5]. Most DSSs are generally designed for specific crops or farmlands; it is difficult to apply them in other crops or the same crop planted in different districts. In view of this, Yang [6] developed a decision support system for flexible irrigation scheduling (DSS-FIS), in which users can modify its input parameters through a software interface to adapt it to various environments. 

The use of DSSs for irrigation ties in implementing a reliable decision method and using decision data sources. Weather forecast information can be a way to estimate the water requirements of the crops [7]. However, this method is unilateral, since it ignores the impact of crop characteristics and spatial variability. Some studies have used sensors and the global positioning system (GPS) to collect information about specific soil and plant conditions for irrigation scheduling. Sui [8] built a wireless sensor network to monitor soil water content and collect weather data. O’Shaughnessy [9] used infrared temperature (IRT) sensor nodes mounted on masts at the edge of each concentric treatment area to measure crop canopy temperature. Morari [10] used time domain transmissometry (TDT) sensors to measure soil moisture content. Since these sensors are immobile, the irrigation decision accuracy depends on the number of sensors in the large-scale planting area or on the truss of the sprinkle irrigation machine, and large-scale deployment of these sensors is not economical for a moving sprinkler irrigation system. Also, the sensors mounted in the field are harmful to the farmland soil ecosystem. 

In recent years, irrigation scheduling could be obtained from some schemes based on various remote sensing imagery platforms. Remote sensing technology has the advantages of good real-time performance and wide coverage on cropland, and makes up for the defects of immobile sensors. The development of aerospace technology makes it possible for irrigation scheduling to use remote sensing imagery platforms, with unmanned aerial vehicles (UAVs) and satellites as common tools. However, satellites can be inhibited by clouds and/or may not be in orbital position during crop development stages [11]. Zhang [12] suggested that canopy temperature from UAV thermal infrared images could be a way to evaluate the crop water stress state. The thermal infrared sensors currently mounted are mostly lightweight and uncooled thermal infrared cameras. Compared to multispectral remote-sensing systems, thermal imagery has the advantage of higher reliability, but the multispectral remote-sensing system has better performance in terms of information acquisition stability and mosaic technology maturity. Meanwhile, canopy temperature measured by thermal infrared camera is easily affected by air temperature and human activity [13]. Recently, many studies use UAV spectral images to mark crop water status through the optimization of the vegetation index (VI) and crop water stress index (CWSI) model [14]. The VI-CWSI model, as the main index of the DSS, is usually used as evidence for irrigation dispatching. Zhang [15] used high-resolution UAV multispectral imagery to map maize water stress status. The crop evapotranspiration model (ET_c_) is also used as evidence for irrigation dispatching. Calera [16] reviewed the crop evapotranspiration model (ET_c_) on optical remote sensing for the assessment of crop water requirements, and demonstrated that ET can be a reliable indicator for irrigation assessment. This approach is based on the Food and Agriculture Organization’s (FAO) method for estimating crop evapotranspiration, in which reference evapotranspiration values are multiplied by crop coefficients (Kc). The coefficient may be derived from multispectral VI. Some approaches have employed signals in the thermal band obtained from remote sensors as inputs for energy balance equations that are solved to estimate ET_c_ [17]. The multispectral VI approach for estimating crop evapotranspiration requires fewer inputs and theoretical background knowledge, and is thus simpler than the energy balance equations approach. In this paper, multispectral VIs were used to calculate crop coefficients.

Water stressed crops manifest symptoms such as leaf wilting, stunted growth, and leaf area reduction [18]. VI can be used to monitor surface vegetation conditions. The structure indices based on visible, near infrared and red-edge bands are more widely used, such as the normalized difference vegetation index (NDVI), soil-adjusted vegetation index (SAVI), enhanced vegetation index (EVI), simple ratio (SR) and green normalized difference vegetation index (GNDVI), and visible atmospherically resistant index (VARI). The NDVI is the most often used, partly due to its “ratio” properties, which enable the NDVI to cancel out a large proportion of the noise caused by changing sun angles, topography, clouds or shadow, and atmospheric conditions [19]. The SAVI was established to improve the sensitivity of the NDVI to soil backgrounds [14]. The EVI has thus been considered a modified NDVI with improved sensitivity to high biomass regions and improved vegetation monitoring capability through a de-coupling of the canopy background signal and a reduction in atmospheric influences [19]. The SR can monitor changes in vegetation cover and works best when vegetation is densely covered [20]. The GNDVI is sensitive to crop pigment changes [21]. The VARI can reduce the effects of illumination and atmospheric conditions [22]. This paper measured reflectance indices within multispectral ranges (NDVI, SAVI, EVI, SR, GNDVI, VARI) to indicate canopy changes due to water stress.

The DSS has been used in many cases. The constraints of some kinds of DSSs widely used in agricultural applications have not been eliminated. Firstly, the output of the DSS is the input information of the control system. The actual amount of irrigation is based on the crop’s water requirement, which is not the only factor in practical irrigation scheduling. The DSS should be combined with a VRI control system. Common methods of control systems include zone control and speed control [23]. Speed control varies the moving speed of the center pivot to accomplish the desired irrigation depth, while the duty-cycle control changes the on-off time of individual sprinklers or groups of sprinklers to achieve the desired irrigation depth. Generally, the VRI control system provides opportunities to apply water to management zones by different moving rates or different solenoid valve duty-cycles. Secondly, the CWSI/K_c_ inversion model (VI-Kc/VI-CWSI) is built for specific districts and crops. Different crops have different optimization VI-Kc/VI-CWSI model [24,25]. Different districts with the same crop also have different VI-Kc/VI-CWSI [26]. The DSS based on the fixed model is unsuitable for other applications. Third, the irrigation amount is the result of a multifactorial decision. Actually, precise measurement of the irrigation requirement is complicated, whose implementation needs a lot of funds and time. 

The concept of a fuzzy system has been used for a realistic decision support model. For the irrigation mission, the interaction between the crop water requirement and irrigation amount is not always accurately defined. Therefore, the fuzzy model can be a viable alternative [27]. The fuzzy system has the characteristics of simple rules and wide applicability [28], and can analyze the inaccurate information and receive irrigation guidance from complex farming sites [29]. So it is easier for users to accept DSSs based on fuzzy logic. 

This study developed a DSS for variable rate irrigation (DSS-VRI) that serviced a center-pivot irrigation system. The function of the DSS integrated image processing and data analysis for UAV remote sensing. By using the DSS, users can easily process the image and obtain a duty-cycle control map of variable irrigation. The specific objectives of this research were:

(i) To develop a widely applicable DSS-VRI, which mainly embodies the user to change some conditional parameters and to construct a partial decision model to work in different cases.

(ii) To achieve expected irrigation amounts in different management areas, by using the duty-cycle control map generated from the DSS-VRI, which combines the UAV multispectral remote sensing system and fuzzy inference system.

## 2. System Description and Operation

**Figure 1:**
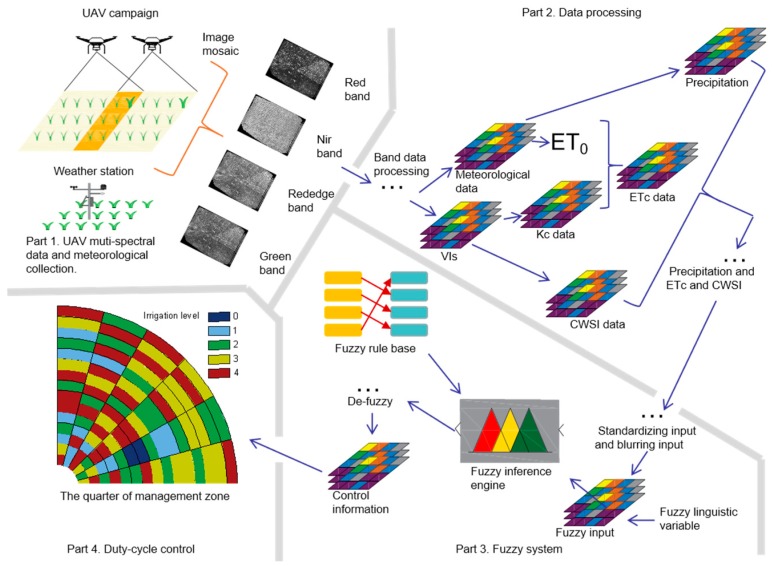
Schematic representation of the decision support system for variable rate irrigation (DSS-VRI). The DSS-VRI operational procedures include four parts. Their functions are as follows. **Part 1** is to provide unmanned aerial vehicle (UAV) multispectral image and meteorological data as input. **Part 2** processes and selects data from **Part 1**, figures out the crop evapotranspiration model (ETc), crop water stress index (CWSI) and precipitation. The data input to the fuzzy system. **Part 3** shows the work flow of the fuzzy system. **Part 4** depicts the duty-cycle control map for a partial management zone.

In this study, a DSS-VRI was designed to make an irrigation scheduling map. The main structure of the DSS-VRI is shown in Figure 1. 

### 2.1. UAV Data Collection and Image Mosaic

**Figure 2:**
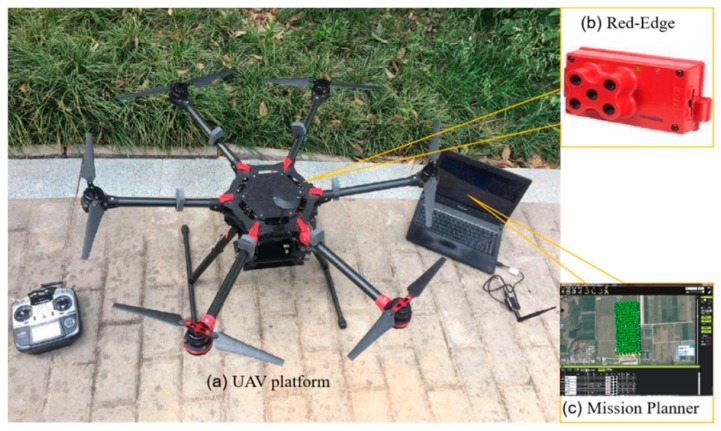
The main elements of the UAV multispectral remote sensing system. (**a**) UAV platform (**b**) RedEdge multispectral camera, and (**c**) the software, Mission Planner.

In this study, the UAV multispectral system was built by the Northwest Agriculture and Forestry University [30]. As shown in Figure 2, this system composed of an unmanned aircraft system, a RedEdge multispectral camera (Micasense, Inc., Washington, USA) and Mission Planner. The unmanned aircraft system included M600 rack and Pixhawk (CUAV, Guangzhou, China) which is an open resource flight control autopilot. It had good performance to take-off and land in different terrain. The UAV takeoff weight, flight time and speed were 6 kg, 18 min and 5 m/s. There were many elements on the unmanned aircraft, such as a gyroscope, accelerometer, magnetometer brushless controllers, etc. Pixhawk integrated all the elements together. Mission Planner is a virtual ground control station for the unmanned aircraft. It connected to Pixhawk by telemetry radios. Users could access the initial setup function to configure the waypoint and the degree of overlap. The technical parameters of RedEdge multispectral camera are shown in Table 1.

Pix4DMapper software was used for image mosaic. Pix4DMapper could stitch image collected from UAV multispectral system and generate five kinds of band image. Pictures spliced by Pix4DMapper could be set to different pixels according to the needs of the user.

Special attention is the arrangement of UAV flight mission. The UAV collected data once a week or twice a week according to actual needs. A UAV flight was conducted between 11:30–12:00 with the multispectral camera. The lens was facing vertically when shooting. The flight height, ground resolution, heading and side overlap were 70 m, 0.05 m/pixel, 80%. Pix4DMapper software platform was used for geometric correction, Gaussian means filtering and multispectral image mosaicking from RedEdge. The entire stitching process took about six hours. The processing period was related to computer performance. Four images (red band, NIR band, blue band, green band) were the input data of DSS-VRI.

### 2.2. Irrigation Decision Model Selected

This work aimed at exhibiting the feasibility of the DSS-VRI to properly manage farmland irrigation. The system is based on the prediction of the crop evapotranspiration and rainfall to obtain fuzzy crop water consumption and replenishment. The crop water stress index (CWSI) was selected to represent the state of water stress. CWSI = 0 indicates no water stress, while CWSI = 1 indicates the most severe stress. The evapotranspiration, rainfall and CWSI were used as three input variables for the fuzzy system to infer the duty-cycle for the central pivot to be reached in order to change the irrigation level within the crop area. The duty cycle is the ratio of the ‘on’ time to the ‘on-off’ period for solenoid valve. Lower duty cycle represents the less amount of water application.

Fuzzy logic can express qualitative knowledge and experience unclear boundaries. It uses the concept of membership function to distinguish fuzzy sets, handle fuzzy relations, and simulate human brains to implement rule-based reasoning. Irrigation problems do not need a high accuracy for water requirement measurement. The integration of fuzzy logic with irrigation planning issues in the field is very effective [31]. Therefore, the method of evapotranspiration, CWSI and precipitation working together through fuzzy inference system can make better decisions for irrigation decision than traditional methods.

#### 2.2.1. Crop Water Evapotranspiration Model (ET_c_) and Crop Water Stress Index (CWSI)

**Figure 3:**
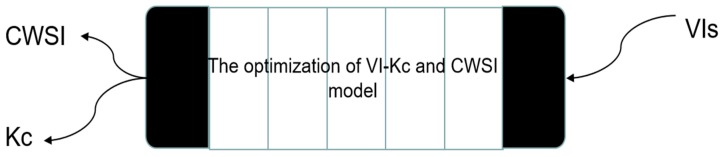
The calculation of the CWSI and ET_c_. The relational formula with the highest correlation coefficient is selected from the established CWSI/Kc inversion model (VI-Kc/VI-CWSI) model, and the optimal model is further established.

The water requirements were obtained by the FAO suggested relation as follows:

(1) $$ET_{C}=K_{C}•ET_{0}$$

where *ET*_0_ is the reference evapotranspiration estimated by FAO Penman–Monteith method that uses data including altitude, latitude, maximum temperature, minimum temperature, mean temperature, average relative humidity, wind speed and sunshine hours. *K_C_* is a crop coefficient obtained by utilizing the VI-Kc model with optimal performance. The CWSI was also obtained from the relational model (VI-CWSI). The models are shown in Figure 3.

To establish a VI-Kc and VI-CWSI model, six VIs (NDVI, EVI, SR, SAVI, GNDVI and VARI) were selected. These VIs were obtained from the UAV based multispectral imagery. Their calculation formulas are shown as follows [14,19,20,21,22]:

(2) NDVI=RNir−RRedRNir+RRed

(3) SAVI=1.5×RNir−RRedRNir+RRed+0.5

(4) EVI=2.5×RNir−RRedRNir+6×RRed−7.5×RBlue+1

(5) SR=RNirRRed

(6) GNDVI=RGreen−RRedRGreen+RRed

(7) VARI=RGreen−RRedRGreen+RRed−RBlue

where *R_Nir_*, *R_Red_*, *R_Blue_* and *R_Green_* are reflectance values of the ground objects in near-infrared, red, blue and green band, respectively.

#### 2.2.2. Fuzzy Logic Model

ET_c_, CWSI and precipitation were used to infer the duty-cycle. Mamdani method was used to implement a fuzzy inference machine. The fuzzy rule of Mamdani can be expressed as follow:

(8) $$\begin{array}{l} R_{i}:\begin{array}{ccc} \begin{array}{ccc} \begin{array}{ccc} if(\begin{array}{ccc} x_{i} & is & X_{i} \end{array}) & and & if(\begin{array}{ccc} y_{i} & is & Y_{i} \end{array}) \end{array} & and & if(\begin{array}{ccc} z_{i} & is & Z_{i} \end{array}) \end{array} & then & n_{i}=Duty-cycle_{i}, \end{array}\\ i=1,...,n \end{array}$$

where *X_i_*, *Y_i_*, *Z_i_* and *Duty -cycle* are time-invariant fuzzy sets, whose membership functions would be defined as a part of system application. Common membership functions are of the follow types: triangular function, ladder function, Gaussian function, bell function, Sigmoid function and Z-type function [32]. This study chose the triangular function and ladder function, because the two functions were simple to use and calculate. Fuzzy inputs were ET_c_, CWSI, precipitation. Fuzzy output was the solenoid valve duty-cycle, which was obtained by defuzzification. In this study, the defuzzification method was centroid.

Fuzzy inputs were defined as three linguistic variables and fuzzy outputs were defined as five linguistic variables: very low (ML), low (L), normal (N), high (H), and very high (MH). According to the basic knowledge of irrigation, 27 fuzzy rules were set for the duty-cycle of the solenoid valve (Table 2). 

These rules were represented with logical operator ‘&’. For example, the first rule is expressed as ‘(ETc==Low) & (Precipitation==Low) & (CWSI==F)=>(Duty-cycle=ML)’, and is interpreted as less rainfall and less evapotranspiration in the coming week, low crop water stress in the current state, so implies lower duty cycle. The duty cycle is the ratio of the ‘on’ time to the ‘on-off’ period. Lower duty cycles represent lower amounts of water application. 

### 2.3. DSS-VRI Software Design and Operation

**Figure 4:**
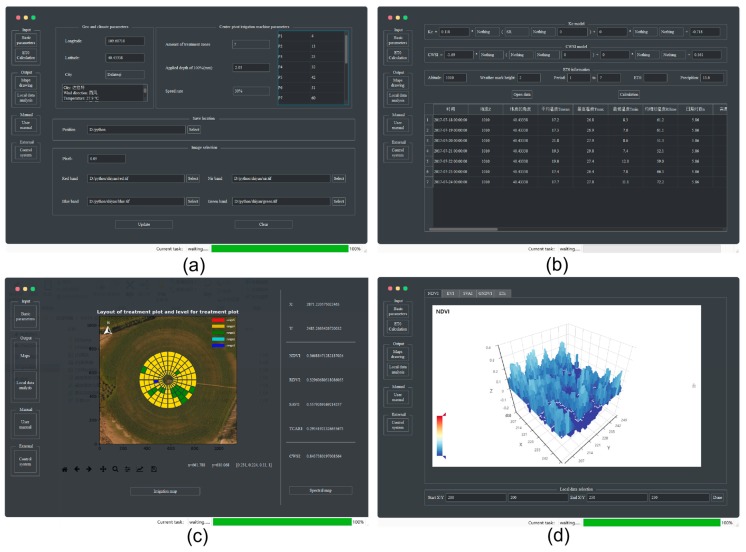
DSS-VRI software interface. (**a**) The basic parameter setting and data input interface. (**b**) CWSI, ET_c_ and precipitation calculation interface. (**c**) The irrigation duty-cycle control map interactive interface. (**d**) The local data analysis interface.

A software was designed to implement the proposed methodology. The application system was programmed in Python (python 3.6.2). The DSS-VRI software generally needs about five minutes to get duty-cycle control map, depending on the input data scale. The user interface was designed and organized into several frames. Figure 4a gives the data input interface, which allow the users to input multispectral image and to set some basic parameters, including geographic coordinates and Moving rate of sprinkler irrigation machine, parameters of zone management, application depth under the 100% moving rate, saving path of duty-cycle control command. Among all parameters, geographic coordinates can be used to find the center position of the machine in remote sensing image. Zone management parameters are used to set the number of electromagnetic valve groups and the distance between the sprinkler center and each management group boundary. Moving speed is related to the maximum crop water requirement. The relationship can be expressed as ‘the more crop water is required, the slower the speed is’. After all parameter values are entered, the user can click the “Update” button to get band information and header file of the image. The interface for calculation of CWSI, ET_c_ and precipitation is shown in Figure 4b. Users can select the VI-Kc model and VI-CWSI model, and enter weather forecast data of the next week to calculate ET0 and rainfall. Figure 4c shows the irrigation duty-cycle control map and NDVI map interface. Users click the “Irrigation map” and “Spectral map” button to get a variable irrigation duty-cycle control map and a NDVI map, respectively. The NDVI map has the information interaction function, which can display VIs and ET_c_ of the mouse location. Figure 4d shows the local data analysis interface, which can assist users to observe local variation of VI by using 3D-bar plot. Figure 4d presents NDVI examples, including the coordinates, NDVI value and spatial distribution. Users click the “User manual” button to get help page and “Control system” to open control system software. In addition, the help page of the DSS-VRI can guide users to learn about the system’s structure and function descriptions.

## 3. Application and Performance Evaluation for System

A 1.13 ha research field located in Zhaojun Town, Dalate Banner, Ordos, Inner Mongolia, China, was taken as an example to demonstrate the DSS-VRI’s feasibility. The whole running period of the DSS-VRI was 6.5 h; 2185 images (five bands) were collected during a single flight in 18 min.

### 3.1. The Study Site Description

**Figure 5:**
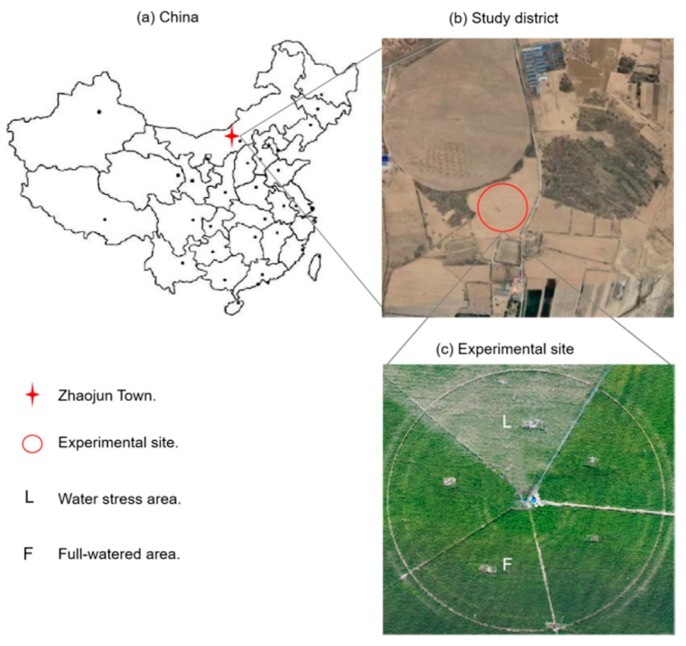
Experimental site description. (**a**) The location of study district. (**b**) The location of experimental site. (**c**) Division of treatment region, water stress area and full-watered area.

The study site is located in the north of China (40°26′0.29″N, 109°36′25.99″E, Elev. 1010 m) (Figure 5). Its climate belongs to the warm temperate zone. Maize (Junkai 918) was the main crop in the study site, planted on 20 May 2017. Maize was planted in east-west oriented rows, spaced at 0.58 m (between rows) and 0.25 m (between columns). The maize emerged on June 1, headed on July 20, and was harvested on September 7 (silage) with 110 days’ lifespan. Natural rainfall in the semi-arid was difficult to meet crop water requirements. The main method of water supply was sprinkler irrigation. On the experiment day, the weather was sunny and the UAV could fly stably under the windy conditions. 

### 3.2. Experimental Design

#### 3.2.1. Water Stress Treatment for Study Site

As shown in Figure 5c, the study field was divided into two treatment regions (TR) with differential irrigation treatment amounts. Water was applied by a center pivot sprinkler (Valmont, NE, USA) equipped with a variable irrigation control system that was developed by our team. During the experimental preparation period (8/14–8/27), irrigation amount in TR F was 12 mm and TR L was 0. Therefore, the TR L maize was in the state of water stress.

#### 3.2.2. Parameter Setting

According to the early experimental results of our team [15,30], the Kc with a different growth stage and different water stress status was calculated by the double crop coefficient method based on the data of corn, soil, and meteorology in the field. Meanwhile, canopy temperature, field air temperature, and relative humidity were used to establish CWSI empirical model. The VI related to crop water stress was derived from the UAV multispectral imagery and used to establish CWSI and Kc inversion models under the weather conditions in Ordos, Inner Mongolia, China. The Kc-VIs relationship (NDVI, SAVI, EVI, SR, GNDVI, VARI) and CWSI-VIs relationship (NDVI, SAVI) were summarized for the periods of middle to late growth. They are shown in Table 3. 

It can be seen from Table 3 that different VIs have different correlations with Kc and CWSI at different growth periods. From the middle to the later growing states, the relevance of the VI-Kc model is ordered from large to small as SR, GNDVI, VARI, NDVI, SAVI, and EVI. The SR of maize in different growth stages had the best correlation with Kc, while SAVI had the best correlation with CWSI than NDVI. In this study, the SR-Kc and SAVI-CWSI model were used to estimate crop coefficients and crop water stress index, respectively.

**Figure 6:**
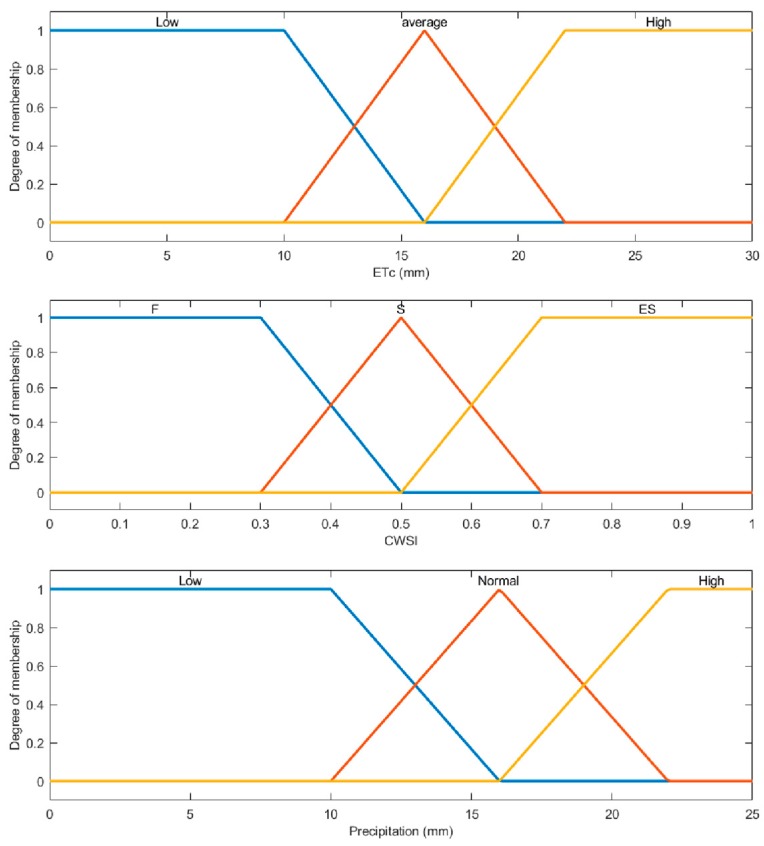
Triangular and ladder membership functions input to the fuzzy system. The linguistic variables are interpreted as follows: “Low” for less rainfall or evapotranspiration in the coming week, “Average and Normal” for normal rainfall and evapotranspiration in the coming week, and “High” for rainy or high evapotranspiration in the coming week. F for crop water sufficient in the current state, S for mild water stress, ES for the most severe stress. The horizontal scales of ETc, CWSI, and precipitation represent evapotranspiration (in mm) in the coming week, the water stress index, and the amount of precipitation (in mm) in the coming week, respectively.

The setting of input membership functions was related to the experimental site. The following threshold was adopted to indicate the water stress severity imposed by the irrigation treatments: CWSI ≤ 0.3 for little to no water stress, 0.3 < CWSI ≤ 0.5 for mild to moderate water stress and CWSI > 0.5 for severe water stress. In the Dalate Banner, the average ET_0_ in the growing season was 2.39 mm/d, and the maximum was 4.83 mm/d [33]. The ET_c_ linguistic variable and membership function were shown in Figure 6. Precipitation was a way to replenish water, and its membership function was the same as ET_c_ in this paper. Its linguistic variables included low precipitation, normal precipitation, and high precipitation.

**Figure 7:**
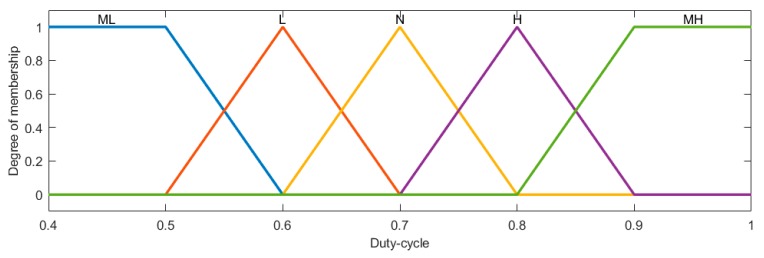
Member functions output from the fuzzy system to the stage of defuzzification. The outputs are built from five linguistic variables: very low (ML), low (L), normal (N), high (H), and very high (MH).

The setting of duty-cycle membership function (Figure 7) was related to the center pivot irrigation system and irrigation requirement. Irrigation depth was calculated as:

(9) $$D_{Rate}=\frac{D_{100\%}}{Rate}$$

where *D_Rate_* is the amount of water application under the moving rate of Rate and *D*_100%_ is under the moving rate of 100%. *D*_100%_ is shown in Table 3.

The model parameters were obtained from different sources. Necessary parameters are shown in Table 4, and can be input through the interface shown in Figure 4a,b.

### 3.3. Results and Discussion

**Figure 8:**
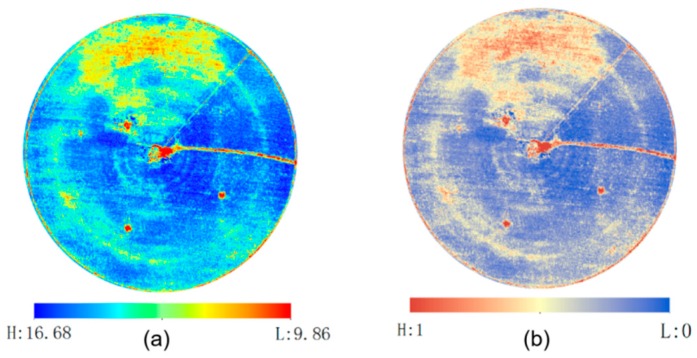
The ET_c_ and CWSI maps. (**a**) ET_c_ map. Blue represents a relatively higher ET_c_, and red lower; (**b**) CWSI map. Blue represents a relatively lower CWSI, and red higher.

The ET_c_ and CWSI map (Figure 8) generated from the DSS-VRI were used as inputs to the fuzzy logic system.

**Figure 9:**
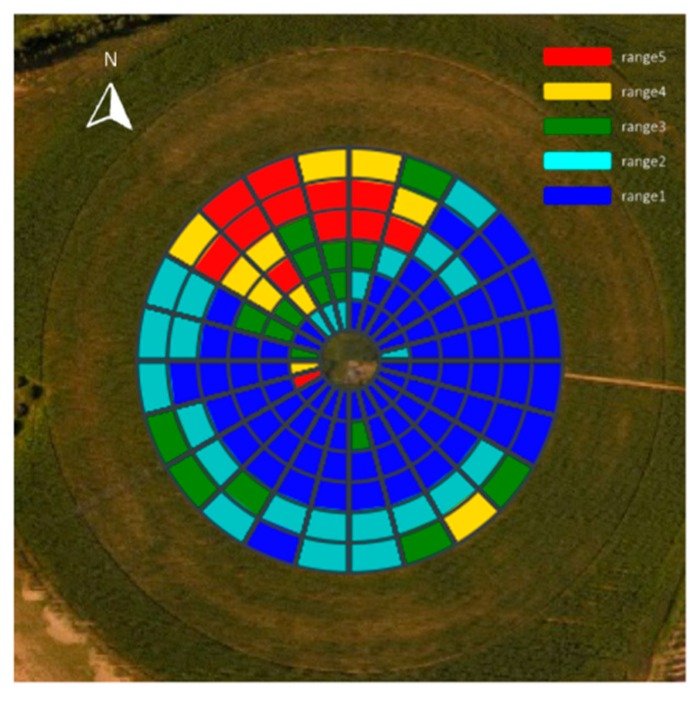
Duty-cycle control map. Corresponding relation of range versus duty-cycle values were range1 to 0.5, range2 to 0.6, range3 to 0.7, range4 to 0.8, and range5 to 0.9.

As shown in the ET_c_ map of Figure 8a, blue represents a relatively higher ET_c_, and red lower. If a crop has insufficient water supply, stomata will close in order to limit water loss through transpiration [34], resulting in a decreased ET_c_. Also, the CWSI can be employed to evaluate water status in plants [35]. When the CWSI is 0, it indicates no water stress; while when the CWSI is 1, this indicates the most severe stress. According to the CWSI map of Figure 8b, blue represents a relatively lower CWSI, and red higher. In this study, the district of lower ET_c_ and higher CWSI matches to water stress treatment regions, consistent with the expected results. After data acquisition and by using preinstalled fuzzy rules, the DSS-VRI fuzzy logic system generates solenoid valve duty-cycle for sub-region of different management. The lower ET_c_ and higher CWSI exhibited in the water stress area (Figure 8), implies more water requirements. In this case, the pivot lateral was configured for six irrigation zone groups. Each irrigation zone was comprised of five sprinkler drop hoses that were hydraulically connected and actuated by a single electronic solenoid valve. The serial number of angle zone for the boundary points at 0°, 15°,..., 345°, and 360°. The distance from each group boundary to center point were 13–23 m, 23–32 m, 32–42 m, 42–51 m, 51–60 m and 60–70 m, respectively. The DSS-VRI combined boundary location information and created a control map with five reference duty-cycle values, as shown in Figure 9.

**Figure 10:**
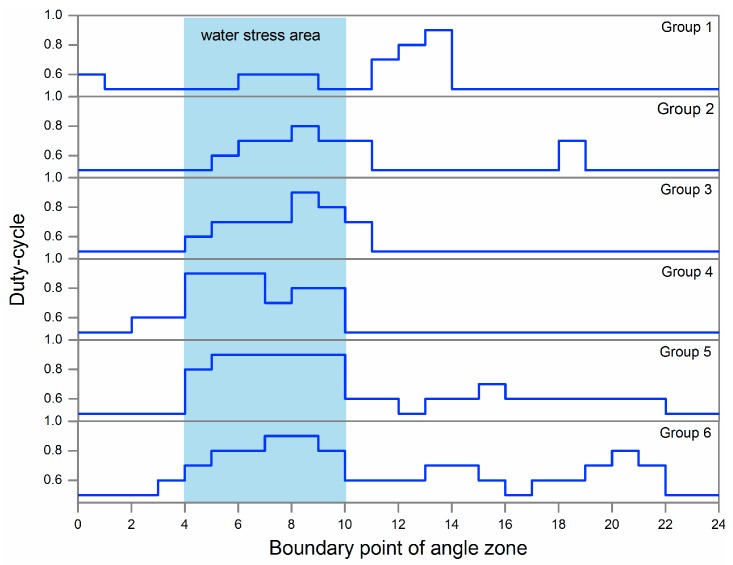
Duty-cycle curve for each management group. The x-axis represents the serial number of angle zone for the boundary points at 0°, 15°, ..., 345°, and 360°. The Y-axis is the duty-cycle. Group 1~Group 6 are the management zones in the radial direction, each group had five solenoid valves.

Figure 10 is the two-dimensional coordinate exhibition of the control map. Under the fixed moving rate of 20%, a higher duty-cycle implies the more irrigation amount, and a lower duty-cycle leads to less irrigation amount. As seen in the Figure, the water stress area was well divided and irrigated with more water. The amount of water application in all directions of Groups 2 to 6 was in line with model expectations. However, for Group 1 with a small radius, it was easy to be affected by adjacent areas’ irrigation, and its stress trait in Figure 10 is not obvious. In addition, its district is between 165°–210° is the working area of underground submersible pump, so the irrigation amount has an obvious fluctuation.

## 4. Conclusion

This study has proposed a decision support system for variable rate irrigation (DDS-VRI). The data inputted to the system were derived from UAV multispectral remote sensing images, and the duty-cycle control map of the solenoid valve was obtained through the fuzzy inference system. To our best knowledge, there is no similar study reported in the previous literature. Fuzzy logic is the core of DSS-VRI. The crop water evapotranspiration model (ETc), precipitation, and the real-time crop water stress index (CWSI) can be used as an effective basis for irrigation management, and they are inputs to the fuzzy inference system. The water supply changes along with the duty cycle when the moving rate is fixed, so the duty cycle can be used as the output of the fuzzy inference system. A user-friendly software interface has been developed to implement the DSS-VRI function. The DSS-VRI output was verified through experiments with realistic irrigation consistent with the model’s expected results.

The DSS-VRI was successful in providing a duty-cycle control map for a central pivot variable rate irrigation system. According to the shape of the management area, the DSS-VRI can also be used for other irrigation systems. For example, for a laterally moving sprinkler system, the DSS-VRI can process remote sensing data through establishing a two-dimensional coordinate system, and obtain a duty-cycle map with a square management area. In general, a broader application of the DSS-VRI primarily depends on the data collection system and fuzzy rules. A multispectral remote sensing system has been used in many cases. Good correlation was demonstrated between crop water state and some multispectral vegetation indexes (VIs). However, performance of multispectral remote sensing on low coverage crops is usually bad. Thus, the reliability of input data sources is the key for future studies to develop a reliable prescription map or control map.

## Figures and Tables

**Table 1:** Parameters of RedEdge multispectral camera.

Parameter	Value
Bands	Blue (475 nm), Green (560 nm), Red (668 nm), Near infrared (Nir) (840 nm), Red-edge (717 nm)
Focal length	5.5 mm (fixed lens)
Angle of view	47.2°
Weight	150 g
Image resolution	1280 × 960 mm

**Table 2:** **The** DSS-VRI fuzzy rule for duty-cycle of solenoid valve.

n	Rule
1	(ETc==Low) & (Precipitation==Low) & (CWSI==F) => (Duty-cycle=ML)
2	(ETc==Low) & (Precipitation==Low) & (CWSI==S) => (Duty-cycle=N)
3	(ETc==Low) & (Precipitation==Low) & (CWSI==ES) => (Duty-cycle=MH)
...	...
13	(ETc==average) & (Precipitation==Normal) & (CWSI==F) => (Duty-cycle=ML)
14	(ETc==average) & (Precipitation==Normal) & (CWSI==S) => (Duty-cycle=N)
15	(ETc==average) & (Precipitation==Normal) & (CWSI==ES) => (Duty-cycle=MH)
...	...
25	(ETc==High) & (Precipitation==Normal) & (CWSI==ES) => (Duty-cycle=MH)
26	(ETc==High) & (Precipitation==High) & (CWSI==S) => (Duty-cycle=L)
27	(ETc==High) & (Precipitation==High) & (CWSI==ES) => (Duty-cycle=N)

Note: “Low” is interpreted as less rainfall or evapotranspiration in the coming week, “average” and “Normal” as normal rainfall and evapotranspiration in the next week, “High” as rainy or high evapotranspiration in the coming week, F as crop water stress in the current state. S as mild water stress, and ES as most severe stress.

**Table 3:** Relationships of vegetation indices with Kc and CWSI for maize from its middle to later growing stages.

Dependent Variable	Vegetation Index	Fitted Formulas	*R* ^2^	*RMSE*
*K_c_*	NDVI	*y* = 6.237*x* − 4.534	0.67	0.1695
	SAVI	*y* = 6.164*x* − 3.016	0.57	0.1926
	EVI	*y* = 3.500*x* − 1.681	0.37	0.2338
	SR	*y* = 0.118*x* − 0.718	0.85	0.1142
	GNDVI	*y* = 4.399*x* − 0.961	0.80	0.1311
	VARI	*y* = 4.266*x* − 0.697	0.71	0.1569
*CWSI*	NDVI	*y* = −1.819*x* + 1.12	0.72	0.046
	SAVI	*y* = −1.69*x* + 0.361	0.81	0.037

**Table 4:** Data input and Parameter setting.

Type	Value	Set time	Source
Coordinate	109.60718E	Fixed	GPS
	40.43338N		
Treatment zone *	24 (0–15°, ..., 345–360°)	Fixed	User
Treatment zone **	6 (4,13,23,32,42,51,60)	2017.8.28	User
Water application depth of 100%	2.05 mm	Fixed	Sprinkler parameters
Speed rate	20%	2017.8.28	Sprinkler parameters
Input image	red, nir, blue and green bands		Remote sensing image
SR-Kc	*y* = 0.118*x* − 0.718	2017.6.11–2017.8.27	Table 3
SAVI-CWSI	*y* = −1.69*x* + 0.361		Table 3
ET_0_	16.3 mm		Meteorological data Meteorological data
Precipitation	13.6 mm		

* Angle counterclockwise direction; ** the radial direction.
